# Tumorigenicity of RTK/RAS in urothelium

**DOI:** 10.18632/oncoscience.188

**Published:** 2015-08-10

**Authors:** Xue-Ru Wu, Cathy Mendelsohn, David J. DeGraff

**Affiliations:** Departments of Urology and Pathology, New York University School of Medicine, Veterans Affairs New York Harbor Healthcare System, Manhattan Campus, New York, NY, USA

**Keywords:** bladder cancer, tumorigenicity, RTK, RAS, oncogene

Although bladder cancer (BC) was the cancer from which the first human oncogene, RAS, was identified, questions persisted over the past 35 years as to whether RAS activation in urothelium was tumorigenic [1]. This was due in large part to the relatively low frequency (~15%) and lack of grade and stage association of RAS mutations in human BC [2]. However, the tide is turning recently in favor of a role for RAS in urothelial tumorigenesis, because of the sheer abundance of the mutations in proteins that act up- and downstream of RAS. For instance, activating mutations of fibroblast growth factor receptor 3 (FGFR3) occur in 45-75% of human BC; those of PI3K and RAF in ~25% and ~8% of human BC, respectively; and those inactivating PTEN in ~8% of human BC [1, 2]. Since most of these mutations are non-overlapping in a given BC, it is not difficult to come to the conclusion that the RTK/RAS signaling pathway is activated in an overwhelming majority of human BC. So, is RTK/RAS pathway activation tumorigenic and, if so, in what context? The recent paper by He et al. [3] and several earlier reports that targeted specific mutations of RTK/RAS pathway components into urothelia of transgenic mice are starting to offer useful clues.

First, the tumorigenicity of activated RTK/RAS components by themselves in urothelium is in general very limited and gene-specific. For instance, expression of a constitutively active kinase mutant of FGFR3 (K644E) in urothelium resulted in normal-appearing epithelia even in aged (18-month old) mice [4]. Expression of a G12V HRAS mutant from its endogenous promoter did not lead to any urothelial abnormality within a year span [5]. Deletion of both but not one allele of PTEN led to urothelial hyperplasia, with only 10% of the mice eventually developing low-grade papillary BC during between 10-20 months [6]. Thus, the growth-promoting potential of activated RTK/RAS pathway varies from component to component, although none seems overly strong. Second, the tumorigenicity of activated RTK/RAS pathway is dosage-dependent. This was best illustrated in transgenic mice expressing an HRAS mutant under the control of a heterologous, Upk2 promoter [7]. While the heterozygous mice consistently developed urothelial hyperplasia before 10 months of age, 100% of the homozygous littermates developed low-grade, papillary BC as early as 3 months and succumbed to obstructive renal failure by 6 months. It appears, therefore, that the magnitude of RAS activation contributes in a major way to RAS-mediated urothelial tumorigenesis. Third, evidence is mounting that the lack of urothelial umorigenicity of RTK/RAS pathway activation had a great deal to do with the multiple compensatory tumor defenses. A range of CDK inhibitors, tumor suppressors, pro-senescence and pro-apoptotic molecules were markedly up-regulated in urothelial cells expressing activated RTK/RAS components [7]. It is conceivable that these failsafe mechanisms serve as effective barriers preventing urothelial tumorigenesis. Finally, urothelial tumorigenesis is an interesting example of context dependence and unique collaborative relationships between oncogenic and tumor-suppressive activities. Case in point, the loss of p16Ink4a and p19Arf, an event found to cooperate with RAS activation in many tissue types to initiate tumors, failed to do so in urothelium [7]. In striking contrast, as recently reported by He and colleagues, the loss of p53 collaborated with activated HRAS to sufficiently induce carcinoma in situ and muscle-invasive BC [3]. Interestingly, the invasive tumors in the compound transgenics expressing the activated HRAS and lacking p53 resemble the “basal” subtype of human BC, including the expression of markers for BC progenitor cells, epithelial-to-mesenchymal transition and squamous differentiation [3]. These findings are of particular clinical significance as the basal-subtype invasive BC in humans, particularly that containing the squamous components, is often resistant to neoadjuvant chemotherapy and carries a high risk of progression to the incurable stage [2, 8].

Based on the existing data from the genetically engineered mice, it is clear that the tumorigenicity of RTK/RAS pathway depends on the oncogenic strengths and the intricate crosstalk of a given RTK/RAS component with specific tumor suppressors. The activation of this pathway can no longer be considered a signature of the low-grade papillary BC pathway as previously thought. Instead, it likely plays a role in the tumorigenesis of both low-grade papillary and high-grade invasive BC pathways, depending on the presence of concomitant genetic alterations. Such divergent partnerships may contribute to the tumorigenesis of the many phenotypic variants that have recently been identified in human BC using whole-genome, multi-platform analyses. Understanding of these intricate relationships will help improve the diagnosis, prognostication and therapy of the various forms of BC.
